# Primary care and health inequality: Difference-in-difference study comparing England and Ontario

**DOI:** 10.1371/journal.pone.0188560

**Published:** 2017-11-28

**Authors:** Richard Cookson, Luke Mondor, Miqdad Asaria, Dionne S. Kringos, Niek S. Klazinga, Walter P. Wodchis

**Affiliations:** 1 Centre for Health Economics, University of York, York, United Kingdom; 2 Institute for Clinical Evaluative Sciences, Toronto, Ontario, Canada; 3 Health System Performance Research Network, Toronto, Ontario, Canada; 4 Academic Medical Centre, University of Amsterdam, Department of Public Health, Amsterdam Public Health Research Institute, Amsterdam, Netherlands; 5 Institute of Health Policy, Management and Evaluation, University of Toronto, Toronto, Ontario, Canada; 6 Institute for Better Health, Trillium Health Partners, Mississauga, Ontario, Canada; Leibniz Institute for Prevention Research and Epidemiology BIPS, GERMANY

## Abstract

**Background:**

It is not known whether equity-oriented primary care investment that seeks to scale up the delivery of effective care in disadvantaged communities can reduce health inequality within high-income settings that have pre-existing universal primary care systems. We provide some non-randomised controlled evidence by comparing health inequality trends between two similar jurisdictions–one of which implemented equity-oriented primary care investment in the mid-to-late 2000s as part of a cross-government strategy for reducing health inequality (England), and one which invested in primary care without any explicit equity objective (Ontario, Canada).

**Methods:**

We analysed whole-population data on 32,482 neighbourhoods (with mean population size of approximately 1,500 people) in England, and 18,961 neighbourhoods (with mean population size of approximately 700 people) in Ontario. We examined trends in mortality amenable to healthcare by decile groups of neighbourhood deprivation within each jurisdiction. We used linear models to estimate absolute and relative gaps in amenable mortality between most and least deprived groups, considering the gradient between these extremes, and evaluated difference-in-difference comparisons between the two jurisdictions.

**Results:**

Inequality trends were comparable in both jurisdictions from 2004–6 but diverged from 2007–11. Compared with Ontario, the absolute gap in amenable mortality in England fell between 2004–6 and 2007–11 by 19.8 per 100,000 population (95% CI: 4.8 to 34.9); and the relative gap in amenable mortality fell by 10 percentage points (95% CI: 1 to 19). The biggest divergence occurred in the most deprived decile group of neighbourhoods.

**Discussion:**

In comparison to Ontario, England succeeded in reducing absolute socioeconomic gaps in mortality amenable to healthcare from 2007 to 2011, and preventing them from growing in relative terms. Equity-oriented primary care reform in England in the mid-to-late 2000s may have helped to reduce socioeconomic inequality in health, though other explanations for this divergence are possible and further research is needed on the specific causal mechanisms.

## Introduction

There are substantial socioeconomic inequalities in health in all high income countries [1], which have persisted in recent decades, and in some cases grown [2,3]. In the United States, for example, one study found a 5 year gap in remaining life expectancy for men aged 50 in 1970 between the top and bottom 10 percent of earners [4]. That gap more than doubled to 12 years by 2000, and for women the gap grew from 3.7 years to 10.1 years.

The World Health Organisation and other public health advocates often claim that public investment in primary care can reduce health inequalities [5–7]. There is some evidence of a link between primary care expenditure and average population health in high income countries [8–10]. However, the most convincing evidence that primary care strengthening can reduce socioeconomic inequality in health has been found in settings in which low income families faced substantial financial barriers to primary care access, including studies in low and middle income countries and a randomised controlled trial in the US in the 1970s [11]. This evidence has limited relevance to today’s high income countries, which all offer free or heavily subsidised primary care to low-income families–including the US, in which nearly 50% of health expenditure is publicly funded compared with the Organisation for Economic Co-operation and Development (OECD) average of 72% in 2012 [12]. Starting from the current state of primary care systems in high-income settings, the potential for using further public investment in primary care to reduce socioeconomic inequalities in health is uncertain.

We address this issue by comparing health inequality trends in two similar high income jurisdictions which invested in primary care in two quite different ways during the mid-to-late-2000s.

England strengthened primary care in an “equity-oriented” way during the mid-to-late 2000s, by making health inequality reduction an explicit policy objective and prioritising investment in scaling up the delivery of effective care for reducing premature mortality in socially disadvantaged populations. By contrast, Ontario strengthened primary care in a non-equity-oriented manner, without any explicit health inequality reduction objective or any sustained effort to prioritise investment in care for socially disadvantaged populations. Both jurisdictions had similar levels and growth rates in national income, social protection and health care expenditure during the period, as detailed in the discussion section. Both also have longstanding equity goals relating to health care access and financing, and have long offered their citizens a relatively generous package of primary care, largely free at the point of use [13,14]. Unlike Ontario, however, England implemented equity-oriented primary care reforms in the mid-to-late 2000s as part of a broader cross-government strategy for reducing health inequality. The cross-government health inequality strategy was announced in 2003, with national health inequality targets set for 2010 [15,16]. However, equity-oriented primary care reform only started in 2006 when reducing inequality in all-age-all-cause mortality was included as a top six priority for the NHS and subject to scrutiny within official NHS policy and planning frameworks in the run up to the 2010 deadline [17] The reforms aimed to re-direct NHS staff time towards scaling up the delivery of effective primary care for preventing premature mortality in disadvantaged adults, in particular drugs to control blood pressure and cholesterol and smoking cessation services. Key components of equity-oriented reform during this period included (1) targeted investment in family medical practices in deprived communities (in particular but not only the centrally funded “Equitable Access to Primary Medical Care” programme announced in 2006 and funded from 2008–10) [18], (2) a knowledge translation programme of tailored advice for local primary care managers from a “National Health Inequality Support Team” on the most effective ways of reducing premature mortality among local disadvantaged populations (2007–9)[17], and (3) the introduction of health checks (2009) [17]. Ontario’s primary care reforms concentrated on increasing payments for primary care team-based practices in the period from 2000–6. Thereafter there were further payments for access and for registering patients with primary care practices [19–21]. In 2004, Ontario also introduced pay-for-performance bonus payments for some screening and specific services to all physicians who participate in rostered models, but these have been shown to have small effects [22]. Notably, Ontario did not introduce any specific policies to address inequity in primary care access or health outcomes. Table 1 summarizes the key primary care reforms recently implemented by both regions until 2011/12, illustrating the policy divergence from 2007 onwards.

**Table 1:** Overview of “equity-oriented” primary care reform in England and contemporaneous primary care reforms in Ontario.

**England**		
**Year**	**Reform**	**Description**
2006	Announcement of targeted investments in primary care supply in underserved areas (funded from 2008) [18]	The “equitable access to primary medical care” programme provided new investment of £250m to support Primary care trusts (PCTs) in establishing at least 100 new family practices in the 25% of PCTs with the poorest provision; and one new FP-led health centre in each PCT in easily accessible locations [18].
	Health inequalities national priority [17]	In 2006, the NHS listed the reduction of health inequalities as a top six NHS priority. From then on, NHS purchasers responsible for their local populations (“Primary Care Trusts”) were required to report on actions taken in their area and there was active regional monitoring of NHS performance against a headline health inequality target to “reduce the inequality gap in all-age all-cause mortality rates” between the most disadvantaged fifth of local areas and the average [17].
2007–09	National guidance and support for chronic care disease management in disadvantaged adults [17]	Guidance was primarily directed towards implementation of effective primary care interventions in disadvantaged adults for secondary prevention of cardiovascular heart disease, diabetes, and other chronic conditions. PCTs and local authorities received tailored support from a ‘national health inequalities support team’ to use an evidence-based ‘Health Inequalities Intervention Tool’ to identify the main causes of death driving local health inequalities and quantify the impact that key interventions could have on local health inequality gaps.
2009	Introduction of NHS Health Checks [17]	The NHS started to implement a programme of vascular risk assessment and management for everyone between the ages of 40–74 who had not already been diagnosed with heart disease, stroke, kidney disease or diabetes. Eligible persons were invited for a check up to assess their risk of these diseases and to offer a tailored package of interventions, as appropriate [17]. Although roll out was slow, with uptake only around 15% of eligible people by 2011, this illustrates one of the many ways in which NHS staff were being encouraged to scale up the delivery of effective primary care interventions for reducing premature mortality in disadvantaged adults [23]
**Ontario**		
**Year** [21]	**Reform**	**Description**
2001/02	Introduction of Family Health Networks (FHN)	FHNs were the first widely available primary care model launched in Ontario that included formal rostering (enrolment) of patients. Physicians practicing in these models are reimbursed through blended capitation, with incentives for set targets (for example, chronic disease management and achieving practice thresholds), and are required to provide after-hour services (evening and weekend). [21].
2003	Introduction of Family Health Groups (FHG)	FHGs maintained many of the same characteristics as FHNs, however, the majority of physician reimbursements are through fee for services. In these models, group practices included a minimum of 3 physicians. The majority (97%) of FHGs were in urban centres [24].
2004/05	Rural-Northern Physician Group Agreement was reached.	This agreement served to increase the delivery of primary care services to rural and northern communities in Ontario, which historically, are in short-supply. Ninety-eight physicians were affected [25].Pay for performance Service Enhancement Payments for five preventative care services: Pap smears, mammograms, flu shot for seniors, toddler immunizations, and colorectal cancer screening; and special payments for services in six areas of care of particular interest to the MOHLTC: payments for obstetrical deliveries, hospital services, palliative care, office procedures, prenatal care, and home visits.
2005	Introduction of the Comprehensive Care Model (CCM)	Intended for solo practices, but with similar provisions as FHG, the CCM provides greater opportunity for higher remuneration for primary care physicians.
	Introduction of Family Health Organizations (FHO)	The FHO model was open to all primary care physicians in Ontario. Its characteristics were similar to the FHN (for example, formal patient enrolment, provision of after-hours services), with a greater number of services and higher capitation rate. By 2010, the FHO was the largest patient enrolment model in the province. Most patients enrolled in this model were from higher income neighbourhoods [21].
	Introduction of Family Health Teams (FHT)	FHTs include an interdisciplinary team of health professionals–including nurses, NPs, midwives, mental health workers such as psychiatrists, nurses and psychologists, kinesiologists, social workers, pharmacists, and nutritionists–that are financially supported by the MOHLTC. Only physicians that are part of FHN or FHO models are eligible to form FHTs [21].
2009	Temporary freeze on all models	The Ministry of Health of Health and Long-term Care (MOHLTC) temporarily froze the hiring of new physicians for group practices (FHN, FHO, FHT, CCM, FHG). At the time, more than two-thirds of physicians practiced in some kind of group model, with more than 9 million patients enrolled into one of the existing primary care programs

This is the first controlled study to provide evidence on this issue. Previous before-after studies have found that socioeconomic gaps in mortality amenable to healthcare in England diminished during the 2000s in absolute terms, but remained constant in proportional terms [26]. Without a control group, however, one cannot conclude that this was due to policy change in England. The observed mortality trends may have occurred in any event due to long-term factors affecting all high income settings, such as the improvements in living conditions and medical technology that helped to reduce mortality from coronary heart disease in all social groups in previous decades [27,28].

## Methods

We conducted a whole-population longitudinal study using health administrative data from England (2011 population: 53.0 million [29]) and Ontario (2011 population: 12.9 million [30]) for fiscal years 2004/05 through 2011/12. In England, datasets included the (1) annual National Health Service (NHS) General and Personal Medical Services workforce census, and (2) Office for National Statistics (ONS) mortality data and mid-year population estimates. In Ontario, datasets included the (1) Registered Persons Database (RPDB), (2) Institute for Clinical Evaluative Sciences Physician Database (IPDB), and (3) Office of the Registrar General (ORGD).

These individual- and practice-level datasets were aggregated into small area geographical units from which we could measure socioeconomic status (SES). In England, there are 32,482 geographical areas defined by the 2001 ‘lower super output areas’ (LSOA), with a mean population size of about 1,500 persons. In Ontario, the nearest approximation to the LSOA, in terms of number of units (n = 18,961) and average population size (700 persons), is the dissemination area (DA). Comparable markers of SES are available for both the LSOA (England) and DA (Ontario): the 2010 Index of Multiple Deprivation (IMD) [31] and the 2006 Ontario Marginalization Material Deprivation Index (ON-Marg) [32], respectively. S1 Table compares the components of the IMD and ON-Marg indices. In each of England and Ontario, small areas were re-aggregated into equally sized decile groups for analysis based on their level of SES and ranked from 1 (most affluent areas) to 10 (most deprived areas). Annual mid-year population estimates were derived for each SES decile, by age and sex, from the ONS (England) and RPDB (Ontario) databases. Less than 2% of Ontario DAs were missing information on SES and thus excluded from the analysis; SES information was available for all English LSOAs.

### Indicators

Our primary outcome of interest was mortality amenable to healthcare, as an indicator of the impact of primary care strengthening on health inequality. We also examined primary care supply to illustrate the differential patterns of primary care investment over time in the two jurisdictions. Both indicators were selected from a broader list because of their population-wide impact and because they were measurable using comparable England and Ontario data sources.

Mortality amenable to healthcare was defined, using the ONS and ORGD datasets, as number of deaths from causes considered avoidable with medical intervention. We considered causes of death (10 groupings of clinical conditions) and the diagnostic criteria as specified by the NHS Outcomes Framework (S2 Table). Following this definition, estimates were restricted to persons aged 0–74 years. To enable comparability over time and between jurisdictions, estimates were directly standardized by age and sex using a weighted (50:50) estimate of the combined 2011 populations of England and Canada (from census data), and scaled to 100,000 persons.

Patients per family physician (i.e. primary care supply) was defined as the population per full-time equivalent primary care physician. In England (NHS data), all primary care physicians were included in the denominator apart from “registrars” (trainees) and “retained doctors” (who work a minimum of four half day sessions per week). In Ontario (IPDB data), we included all active physicians with specialty in General or Family Practice (GP/FP), GP/Emergency Medicine (excluding those who provided >50% of their services in the emergency department) or Community Medicine/Public Health. In each jurisdiction, these physicians comprise the majority of the primary care physician workforce. Annual population estimates for each SES decile were adjusted by age and sex, according to the 2007 revision of the Carr-Hill primary care workload adjustment [33]. This provides weighting to patients according to the frequency of healthcare consultations by age and sex groups.

### Inequality measures

We measured inequalities in amenable mortality in each year of the study period and in each jurisdiction using the slope index of inequality (SII) and relative index of inequality (RII). These measures have been described extensively elsewhere [34–36]. In brief, the SII represents the linear regression coefficient that shows the association between the age-sex adjusted indicator estimate of each SES decile group (outcome) and the cumulative percent of the population ranked by SES (independent variable). The SII can be interpreted as the absolute difference in the outcome between the (hypothetically) most deprived small areas and the most affluent small areas. The RII was calculated by dividing the SII by the population mean. The RII represents the proportionate gap between the most and least deprived small areas, relative to the population mean. For both the SII and RII, large positive values reflect high levels of inequality with worse outcomes in the most deprived small areas.

### Statistical analyses

Average annual changes in amenable mortality were assessed using decile group-level data, for the periods before (2004–6) and after (2007–11) the differential implementation of equity-oriented primary care strengthening in England. These periods were selected to allow for a short lag after the initial implementation of reform in England in 2006 (Table 1). Due to data privacy reasons, indicator estimates for each SES decile group had to be derived separately for each of England and Ontario. We pooled aggregate data from each jurisdiction and used a difference-in-differences (DID) approach based on linear regression to quantify the impact of equity-oriented primary care strengthening in England relative to changes in Ontario. Separate models were fitted for absolute (SII) and relative (RII) inequality measures. For interpretability, we also fitted models specific to the full population (age-sex standardised) amenable mortality rate, and rates in least (D01) and most (D10) deprived decile groups. Each fitted model included independent dichotomous variables indicating the country (England vs. Ontario), the time period (2007/08-2011/12 vs. 2004/05-2006/07), and a 2-way interaction term between these variables, the DID estimator.

Difference-in-differences assume parallel trends. To verify this assumption we tested for statistical differences in pre-2007/08 annual trends in amenable mortality rates (overall, and in the most and least deprived groups) and inequalities (SII and RII) between England and Ontario using linear regression models with an interaction term between jurisdiction and year. Parallel trends were confirmed (p>0.05 for each interaction) for all planned analyses. The study received data governance approval from the Health and Social Care Information Centre (England) and approval from the Research Ethics Board at Sunnybrook Health Sciences Centre (Ontario).

## Results

### Trends in primary care supply

**Fig 1:**
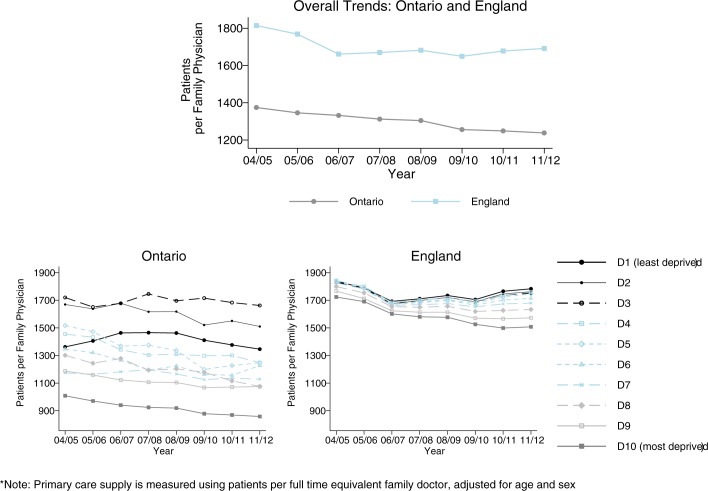
Trends in primary care supply in England and Ontario, overall (top) and by socioeconomic decile (bottom) (whole population means).

Over the course of the study period both Ontario and England increased the primary care workforce per head of population, but there were fewer patients per family physician in Ontario than in England. After 2006/7, overall primary care supply increased more rapidly in Ontario (Fig 1, top panel), but England shifted supply in a more equity-oriented direction (Fig 1, bottom panel). Across SES groups (Fig 1, bottom panel), more deprived neighbourhoods tended to have fewer patients per family physician, though this socio-economic gradient was wider and not entirely monotonic in Ontario. This “reverse” gradient in primary care supply favouring disadvantaged neighbourhoods reflects the greater burden of illness and need for primary care in deprived communities, as we were unable to adjust for this using comparable morbidity data across both jurisdictions. In Ontario this “reverse” social gradient remained moderately stable, whereas in England it widened each year from 2006/7 onwards, reflecting the national policy to recruit new family doctors in deprived communities.

### Trends in amenable mortality

**Fig 2:**
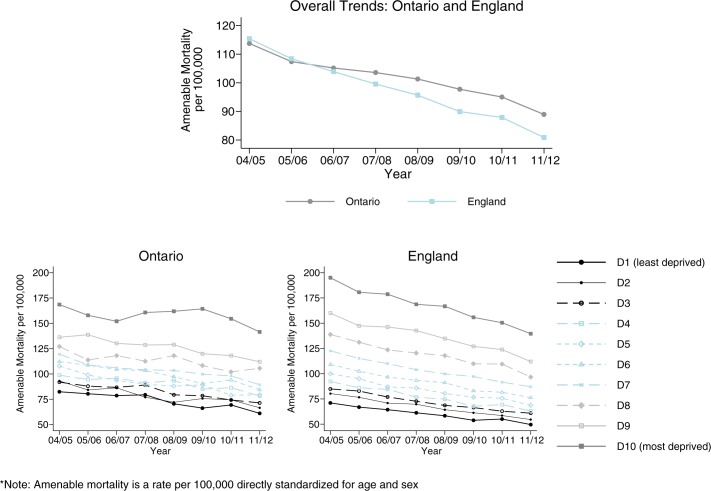
Trends in amenable mortality in England and Ontario, overall (top) and by socioeconomic decile (bottom) (whole population means).

Mortality rates from causes considered amenable to health care decreased in both Ontario and England from 2004/5 to 2011/12 (Fig 2, top panel). Although overall rates in both jurisdictions trended downwards, improvements were not statistically significant in the post-reform period due to wide variability in annual estimates at the decile level (S3 Table). These data are shown in Fig 2 (bottom panel): a socio-economic gradient was consistently observed for both jurisdictions in each year of the study period, with higher mortality in more deprived areas. Notably, mortality among deprived groups fell more rapidly in England compared to Ontario in the post-2006/7 period. In particular, among the most deprived decile group, amenable mortality fell substantially and significantly in England by an annual average of 7.5 deaths per 100,000 population (95% CI: 4.8 to 10.1, S3 Table). The decline in Ontario’s most deprived group, in contrast, was not statistically significant (4.6 fewer deaths per 100,000 population, 95% CI: -2.0 to 11.1, S3 Table).

### Changes in inequalities in amenable mortality and the impact of policy implementation

**Fig 3:**
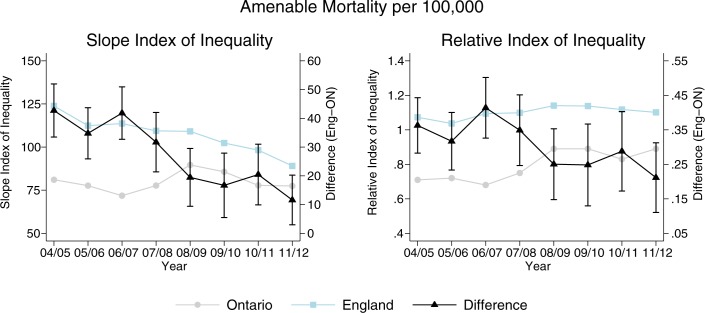
Differential health inequality trends in England and Ontario.

Measured levels of inequality were larger in England than Ontario throughout the study period for amenable mortality (Fig 3). However, larger “pro-poor” improvements (i.e., a reduction in the social gradient in amenable mortality) from 2006 onwards were observed in England as compared to Ontario. The difference in the modelled absolute inequality gap between England and Ontario narrowed over the study period, due largely to a greater reduction in absolute inequality (SII) in England beginning after 2006/7. In relative terms (RII), the social gradient in mortality increased in Ontario relative to the population mean, while it remained relatively constant in England.

Table 2 highlights the results from DID analyses, which quantify the differential change in rates in amenable mortality for each jurisdiction overall, in the least and most deprived groups, and in absolute and relative inequalities between England and Ontario before (2004–6) and after (2007–11) the introduction of England’s equity-oriented health care reform. The change in overall rates of amenable mortality was comparable between England and Ontario pre- vs. post-2007/08 (DID estimator: 7.0, 95% CI: -26.1 to 12.1), as well as between the least deprived groups (DID estimator: -0.7, 95% CI: -11.7 to 10.4). However, large and statistically significant improvements pre- vs. post-2007/8 were found in the most deprived areas in England, relative to Ontario (DID estimator: -25.6, -48.3 to -3.0). This difference largely contributed to the statistically significant decreases in absolute inequalities (DID estimator for SII: -19.9, 95% CI: -34.9 to -4.8) and relative inequalities (DID estimator for RII: -0.10, 95% CI: -0.19 to -0.01) in England compared to Ontario.

**Table 2:** Difference-in-difference comparison between 2004–6 and 2007–11.

	Overall Mean	D01 (Least Deprived SES Decile)	D10 (Most Deprived SES Decile)	Slope index of Inequality (SII)	Relative Index of Inequality (RII)
**Amenable Mortality per 100,000**					
Ontario 2004–6	108.8 (99.9, 117.6)	80.5 (75.8, 85.2)	159.5 (138.7, 180.2)	76.8 (65.2, 88.5)	0.70 (0.65, 0.76)
England 2004–6	109.2 (95.8, 122.7)	67.5 (59.1, 75.9)	184.8 (162.6, 207.0)	116.6 (101.1, 132.2)	1.07 (1.00–1.14)
^1^ Difference	0.5 (-15.3, 16.2)	-13.0 (-19.2, -6.8)	25.3 (5.7, 44.9)	39.9 (27.4, 52.4)	0.36 (0.31, 0.42)
Ontario 2007–11	97.3 (89.9, 104.7)	69.3 (61.0, 77.7)	156.6 (145.2, 168.0)	81.7 (74.7, 88.7)	0.85 (0.77, 0.93)
England 2007–11	90.8 (81.8, 99.8)	55.7 (50.2, 61.2)	156.3 (141.3, 171.2)	101.6 (91.1, 112.1)	1.12 (1.10, 1.14)
^1^ Difference	-6.5 (-18.0, 5.0)	-13.7 (-22.0, -5.4)	-0.3 (-15.9, 15.3)	20.0 (9.5, 30.5)	0.27 (0.20, 0.34)
^***2***^ ***Difference-in-Differences***	***-7*.*0 (-26*.*1*, *12*.*1)***	***-0*.*7 (-11*.*7*, *10*.*4)***	*-25*.*6 (-48*.*3*, *-3*.0)*	*-19*.*8 (-34*.*9*, *-4*.8)*	*-0*.*10 (-0*.*19*, *-0*.01)*

Notes

^1^ Difference value = England–Ontario, for that time period

^2^ Difference-in-Differences Estimator = (England2007-11—England2004-6)—(Ontario2007-11—Ontario2004-6)

* Denotes statistically significant difference (p<0.05)

## Discussion

### Main findings

Unlike Ontario, England embarked upon a sustained programme of equity-oriented primary care strengthening in the mid to late 2000s as part of the world’s first cross-government strategy for tackling health inequality (Table 1) [15,16]. According to an independent review, the policy priority given by the NHS and other public services to the English health inequality strategy peaked during 2007–9, as the deadline approached for meeting the central government health inequality targets in 2010 [37].

We found that inequality trends in mortality amenable to health care in England and Ontario were comparable from 2004–6 but diverged from 2007–11, with Ontario experiencing less favourable inequality trends in the latter period in terms of both absolute and relative gaps between socioeconomic groups. In terms of absolute gaps, England improved from 2007–11 while Ontario slightly deteriorated. The mean inequality gap in England fell from 116.6 deaths per 100,000 in 2004–6 to 101.6 per 100,000 in 2007–11, after adjustment for evolving differences in the age and sex profiles of the English and Ontarian populations. We estimate that, if England had followed the same path as Ontario, the gap would have risen slightly rather than falling, resulting in an increase of 19.8 deaths per 100,000 (confidence interval 4.8 to 34.9) in the absolute gap in England from 2007–11. A similar divergence was observed in terms of relative inequality gaps, although in this case the story is one of England deteriorating less rapidly than Ontario. Relative inequality gaps are a tougher benchmark in this context, since they are measured as a proportion of mean mortality, which was declining in both jurisdictions during both periods. In England from 2007–11, the inequality gap relative to the mean was 1.12. We estimate that this would have been 10 percentage points higher in 2007–10 (CI 1 to 19) if England had followed the same path as Ontario.

The biggest divergence was observed in the most deprived tenth of neighbourhoods: amenable mortality fell considerably further in England than Ontario between the two periods, by 25.6 deaths per 100,000 population (CI 3.0 to 48.3).

In raw terms, the differences in the level of primary care supply between the highest and lowest deciles of deprivation increased in England with the greatest increases observed in the most deprived geographic areas. Assuming that need is greater in areas of high deprivation [38], we interpret this as a reduction in inequity in the sense of deviation from equal access and outcomes for equal need. This interpretation follows the inverse care law originally advanced by Hart [39] and as described by Watt and others as circumstances where the same primary care resources achieve lesser impact in deprived areas [40,41].

### Comparison with previous studies

Uncontrolled observational studies have found absolute inequality reductions in England between 2004 and 2011 in both primary care supply [38] and amenable mortality [26], and particularly in mortality from cardiovascular diseases [42]. However, these studies were unable to determine whether this trend would have occurred in any event in the absence of primary care strengthening and cross-government action on health inequality. For example, coronary heart disease (CHD) mortality has been falling in all social groups in Great Britain for decades, resulting in falling absolute inequality gaps between social groups since at least 1994 [27].

### Limitations

The main limitation of this study is its reliance on the assumption that Ontario is a useful counterfactual model of what would have occurred in England in the absence of equity-oriented primary care reforms. This assumption may be questioned by pointing to other differences between the two jurisdictions that may have influenced amenable mortality trends during the 2000s, such as differences in wider social and economic trends that may have influenced health behaviour and mortality in disadvantaged communities.

To help defend our assumption, we present some OECD data on comparative trends in some of the social determinants of health in Table 3. These are country-level data relating to the broader populations of the United Kingdom and Canada rather than England and Ontario, but are more comparable than jurisdiction-specific data. They show similar trends in economic growth, economic inequality, social protection, and health care expenditure. The main differences are that Canada experienced slightly faster economic growth than the UK throughout this period, and was slightly more successful in preventing a rise in economic inequality. However, both of these differences would be expected to reduce both overall mortality and socioeconomic inequality in mortality in Ontario, and so cannot explain the opposite trends that we have found.

**Table 3:** Comparative trends in social determinants of health.

	2004	2005	2006	2007	2008	2009	2010	2011
**Gross national income per capita (US$ PPP)** [43]								
UK	33723	35466	37332	37921	37898	36503	36350	37038
Canada	32922	35310	37218	38647	39498	38054	39307	40808
Canada Difference (%)	-2.3	-0.4	-0.3	1.9	4.2	4.2	8.1	10.2
**Income inequality (Gini coefficient)** [44] ^1^								
UK	0.331	0.350	0.354	0.361	0.359	0.362	0.341	0.344
Canada	0.322	0.316	0.317	0.318	0.321	0.320	0.319	0.315
Canada Difference	-0.009	-0.034	-0.037	-0.043	-0.038	-0.042	-0.022	-0.029
**Social protection expenditure per capita (US$ PPP)** [45]								
UK	6620	6787	6877	7045	7379	7814	7516	7475
Canada	5805	5866	6005	6015	6191	6621	6535	6432
Canada Difference (%)	-12.3	-13.6	-12.7	-14.6	-16.1	-15.3	-13.1	-14.0
**Health care expenditure per capita (US$ PPP)** [46] ^2^								
UK	2467	2569	2672	2743	2811	2956	2918	2915
Canada	3224	3282	3392	3463	3523	3769	3843	3795
Canada Difference (%)	30.7	27.8	27.0	26.3	25.3	27.5	31.7	30.2

Notes

^1^ Gini coefficient (disposable income, post taxes and transfers) based on the new OECD income definition since 2012 (exception: England 2004 estimate)

^2^ Current expenditure on health care per capita, constant prices, constant PPPs, OECD base year

A second limitation is that we cannot pinpoint which specific elements of equity-oriented primary care strengthening caused what effects on which mortality disease categories and with what time lag. To support our claim that equity-oriented primary care strengthening in England peaked from 2007–9, we have presented illustrative evidence that England achieved substantial reductions in socioeconomic inequities in family doctor workforce from 2007–9 that were not delivered in Ontario. However, we were not able to compare inequity trends between the two jurisdictions in other important aspects of primary care supply, such as nurse practitioners, or in the use of effective primary care for preventing premature mortality in disadvantaged adults, in particular drugs to control blood pressure and cholesterol and smoking cessation services. Changes in family doctor supply were just one element of a much broader package of equity-oriented primary care strengthening, and so we cannot conclude that the family doctor workforce was the primary causal factor behind the observed differential trends in mortality inequalities between England and Ontario. It is plausible, however, that primary care can have important impacts on amenable mortality–for example, a recent modelling study estimated that around 50% of the fall in CHD mortality in England in the 2000s was due to improved primary care treatment [47].

## Conclusions

Unlike Ontario, England made reducing health inequality a high priority for the healthcare system during the mid-to-late 2000s, and re-directed staff time towards scaling up the delivery of effective care for preventing premature mortality in disadvantaged adults. The divergent trends in mortality amenable to healthcare between England and Ontario from 2007 to 2011 suggest that without this sustained policy action in England, absolute inequality gaps would not have fallen and relative gaps would have increased more rapidly. We therefore conclude that equity-oriented primary care reform in England in the mid-to-late 2000s may have helped to reduce socioeconomic inequality in health, or at least in helping to ameliorate growing health inequalities. However, since this was a non-random study we cannot conclude definitively that the observed differential trends between England and Ontario were caused by “equity-oriented” reform rather than other differences between the two jurisdictions. Furthermore, we have not identified the specific causal pathways that led to these differential trends, so cannot tell which components of reform had which effects on mortality.

### Future research

Further research is needed to disentangle the causal mechanisms underpinning the differential mortality trends that we have observed between England and Ontario, to give policy makers and clinicians a clearer idea of which specific elements of primary care strengthening are particularly important for tackling health inequality. In particular, research using longitudinal individual level data is needed to disentangle the inequality impacts on different categories of amenable mortality (disease-specific causes of death), and on inequality trends in the use of different types of primary care treatment, referral, and medical advice. Our analysis of overall mortality is indicative of differences, but does not specify which diseases have seen the greatest changes in mortality. Future research will also need to allow for differential trends by socioeconomic group in risk factors such as body mass index, hypertension and lipid levels, and health behaviours such as smoking, diet, and exercise, which may act both as confounding factors and also as mediating factors which are themselves partly caused by primary care treatment, referral, and advice. Furthermore, our comparative trend analyses should be extended to different countries and repeated with data over a longer and more recent period of time become available to analyse more recent investments and policies related to primary care and inequity.

## Supporting information

S1 Table Comparison of neighbourhood deprivation measures in England and Ontario. (DOCX)

S2 Table Causes of death considered amenable to healthcare. (DOCX)

S3 Table Pre- and Post-2007/8 trend analyses, results. (DOCX)

S1 File Aggregate data. (XLSX)

## Abbreviations

CHD: Coronary heart disease
DA: Dissemination area
DID: Difference-in-differences
IMD: Index of Multiple Deprivation
IPDB: ICES Physician Database
LSOA: Lower super output areas
NHS: National Health Service
ON-MARG: Ontario marginalization (material deprivation) index
ONS: Office for National Statistics
ORGD: Office of the Registrar General
RPDB: Registered Persons Database
RII: relative index of inequality
SES: Socio-economic status
SII: slope index of inequality
