# Supplementary material for: Primary care and health inequality: Difference-in-difference study comparing England and Ontario
Source: PLoS One. 2017 Nov 28;12(11):e0188560. doi: 10.1371/journal.pone.0188560 (PMC5705159; doi:10.1371/journal.pone.0188560)
Supplement: S1 Table — (DOCX) [file pone.0188560.s001.docx]

**S1 Table: Comparison of neighbourhood deprivation measures in England and Ontario**

| English Deprivation Index^1^: Domains  (Most domains are composites of multiple indicators) | Ontario Marginalization Index^2^, Material Deprivation Index: Dimensions |
| --- | --- |
| 1. Income – proportion of the population living in low-income households 2. Employment 3. Health and Disability 4. Education, Skills and Training 5. Barriers to Housing and Services 6. Crime 7. Living Environment | 1. Proportion of the population considered low-income 2. Proportion of the population aged 15+ who are unemployed 3. Proportion of the population receiving government transfer payments 4. Proportion of the population aged 20+ without a high-school diploma 5. Proportion of households living in dwellings that are in need of major repair 6. n/a 7. Proportion of families who are lone parent families |

*Sources*:

^1^ English Indices of Deprivation 2010 [cited 24 March 2016] Retrieved from <https://data.gov.uk/dataset/index-of-multiple-deprivation>

^1^ English Indices of Deprivation 2010 [cited 24 March 2016] Retrieved from https://www.gov.uk/government/statistics/english-indices-of-deprivation-2010

^2^ Matheson, F. I., Dunn, J. R., Smith, K. L. W., Moineddin, R., & Glazier, R. H. (2012). Development of the Canadian Marginalization Index: a new tool for the study of inequality. *Canadian Journal of Public Health = Revue Canadienne De Santé Publique*, *103*(8 Suppl 2), S12–6.

^2^ Matheson, F. I., Dunn, J. R., Smith, K. L., Moineddin, R., & Glazier, R. H. (2012). *ON-Marg: Ontario Marginalization Index User Guide version 1.0*. [cited 24 Mar 2016] Retrieved from http://www.torontohealthprofiles.ca/onmarg/userguide_data/ON-Marg_user_guide_1.0_FINAL_MAY2012.pdf
