# Supplementary material for: Primary care and health inequality: Difference-in-difference study comparing England and Ontario
Source: PLoS One. 2017 Nov 28;12(11):e0188560. doi: 10.1371/journal.pone.0188560 (PMC5705159; doi:10.1371/journal.pone.0188560)
Supplement: S2 Table — (DOCX) [file pone.0188560.s002.docx]

**S2 Table: Causes of death considered amenable to healthcare**

| **Condition group and cause** | **ICD–10-WHO codes** | **Ages included** |
| --- | --- | --- |
| **Infections** | | |
| Intestinal infectious disease | A00 – A09 | 0-14 |
| Tuberculosis | A15–A19, B90 | 0–74 |
| Other infections (diphtheria, other tetanus, acute poliomyelitis) | A35 – A36, A80 | 0-74 |
| Whooping cough | A37 | 0-14 |
| Selected invasive bacterial and protozoal infections | A38–A41, A46, A48.1, B50–B54, G00, G03, J02, L03 | 0–74 |
| Measles | B05 | 1-14 |
| Hepatitis C | B17.1, B18.2 | 0-74 |
| HIV/AIDS | B20-B24 | 0-74 |
| **Neoplasms** | | |
| Malignant neoplasm of colon and rectum | C18–C21 | 0–74 |
| Malignant melanoma of skin | C43 | 0–74 |
| Other malignant neoplasms of skin | C44 | 0-74 |
| Malignant neoplasm of breast | C50 | 0–74 |
| Malignant neoplasm of cervix uteri | C53 | 0–74 |
| Malignant neoplasms of corpus uteri and uterus unspecified | C54 – C55 | 0-44 |
| Malignant neoplasm of testis | C62 | 0-74 |
| Malignant neoplasm of bladder | C67 | 0–74 |
| Malignant neoplasm of thyroid gland | C73 | 0–74 |
| Hodgkin’s disease | C81 | 0–74 |
| Leukaemia | C91, C92.0 | 0–44 |
| Benign neoplasms | D10–D36 | 0–74 |
| **Nutritional, endocrine and metabolic** | | |
| Disorders of thyroid gland | E00 – E07 | 0 – 74 |
| Diabetes mellitus | E10–E14 | 0–49 |
| **Neurological disorders** | | |
| Epilepsy and status epilepticus | G40–G41 | 0–74 |
| **Cardiovascular diseases (CVD)** | | |
| Rheumatic and other valvular heart disease | I01–I09 | 0–74 |
| Hypertensive diseases | I10–I15 | 0–74 |
| Ischaemic heart disease | I20–I25 | 0–74 |
| Cerebrovascular diseases | I60–I69 | 0–74 |
| **Respiratory diseases** | | |
| Pneumonia | J12–J18 | 0–74 |
| Other respiratory | J00-J08, J20- J39, J47-J99 | 1-14 |
| Gastric and duodenal ulcer | K25–K28 | 0–74 |
| **Genitourinary disorders** | | |
| Nephritis and nephrosis | N00–N07, N17– N19, N25-N27 | 0–74 |
| Obstructive uropathy & prostatic hyperplasia | N13, N20–N21, N35, N40, N99.1 | 0–74 |
| **Maternal & infant** | | |
| Complications of perinatal period | P00–P96, A33 | 0-74 |
| Congenital malformations, deformations and chromosomal anomalies | Q00–Q99 | 0–74 |
| Pregnancy, childbirth and the puerperium | O00 – O99 | 0-74 |
| **Injuries** | | |
| Misadventures to patients during surgical and medical care | Y60–Y69, Y83– Y84 | 0-74 |

*Source:*

The NHS Outcomes Framework 2012/13: Technical Appendix. [cited 24 Mar 2016] Retrieved from: <https://www.gov.uk/government/uploads/system/uploads/attachment_data/file/213713/dh_131721.pdf>
