# Supplementary material for: Primary care and health inequality: Difference-in-difference study comparing England and Ontario
Source: PLoS One. 2017 Nov 28;12(11):e0188560. doi: 10.1371/journal.pone.0188560 (PMC5705159; doi:10.1371/journal.pone.0188560)
Supplement: S3 Table — (DOCX) [file pone.0188560.s003.docx]

**S3 Table: Pre- and Post-2007/8 annual change in indicators**

|  | **Mean** | **D01 (Least Deprived Group)** | **D10 (Most Deprived Group)** |
| --- | --- | --- | --- |
| **Patients per Family Physician** | | | |
| **Ontario** |  |  |  |
| 2004-6 | -21.2 (-122.1, 79.8) | 50.7 (-2.8, 104.3) | -34.1 (-60.4, -7.7)* |
| 2007-11 | -20.4 (-66.8, 26.0) | -32.5 (-45.2, 19.8)* | -18.2 (-28.1, -8.3)* |
| **England** |  |  |  |
| 2004-6 | -76.6 (-93.9, -59.2)* | -72.6 (-248.3, 103.2) | -61.0 (-265.4, 143.5) |
| 2007-11 | 3.9 (-9.9, 17.7) | 17.6 (-5.1, 40.3) | -22.3 (-39.9, -4.8)* |
| **Amenable Mortality per 100,000 population** | | | |
| **Ontario** |  |  |  |
| 2004-6 | -4.3 (-15.2, 6.6) | -1.9 (-2.4, -1.4)* | -8.25 (-25.5, 9.0) |
| 2007-11 | -3.6 (-8.7, 1.6) | -3.8 (-7.3, -0.2)* | -4.6 (-11.1, 2.0) |
| **England** |  |  |  |
| 2004-6 | -5.8 (-22.5, 10.9) | -3.3 (-9.5, 2.8) | -8.2 (-54.0, 37.7) |
| 2007-11 | -4.5 (-10.8, 1.8) | -2.7 (-4.3, -1.0)* | -7.5 (-10.1, -4.8)* |

* Denotes statistically significant difference (p<0.05) from linear regression analysis
